# A Physiologically Based Pharmacokinetic Model to Predict the Impact of Metabolic Changes Associated with Metabolic Associated Fatty Liver Disease on Drug Exposure

**DOI:** 10.3390/ijms231911751

**Published:** 2022-10-04

**Authors:** Elise M. Newman, Andrew Rowland

**Affiliations:** 1 College of Medicine and Public Health, Flinders University, Bedford Park, Adelaide 5042, Australia; 2 Beat Cancer Fellow, Cancer Council South Australia, Adelaide 5042, Australia

**Keywords:** pharmacokinetic model, liver disease, non-alcoholic fatty liver disease (NAFLD), metabolism, simulation

## Abstract

Metabolic associated fatty liver disease (MAFLD) is the most common chronic liver disease, with an estimated prevalence of between 20 and 30% worldwide. Observational data supported by in vitro and pre-clinical animal models of MAFLD suggest meaningful differences in drug disposition in MAFLD patients. This study aimed to build a physiologically based pharmacokinetic (PBPK) model reflecting observed changes in physiological and molecular parameters relevant to drug disposition that are associated with MAFLD. A comprehensive literature review and meta-analysis was conducted to identify all studies describing in vivo physiological changes along with in vitro and pre-clinical model changes in CYP 1A2, 2C9, 2C19, 2D6 and 3A4 protein abundance associated with MAFLD. A MAFLD population profile was constructed in Simcyp (version 19.1) by adapting demographic and physiological covariates from the Sim-Healthy population profile based on a meta-analysis of observed data from the published literature. Simulations demonstrated that single dose and steady state area under the plasma concentration time curve (AUC) for caffeine, clozapine, omeprazole, metoprolol, dextromethorphan and midazolam, but not s-warfarin or rosiglitazone, were increased by >20% in the MAFLD population compared to the healthy control population. These findings indicate that MAFLD patients are likely to be experience meaningfully higher exposure to drugs that are primarily metabolized by CYP 1A2, 2C19, 2D6 and 3A4, but not CYP2C9. Closer monitoring of MAFLD patients using drugs primarily cleared by CYP 1A2, 2C19 and 3A4 is warranted as reduced metabolic activity and increased drug exposure are likely to result in an increased incidence of toxicity in this population.

## 1. Introduction

Non-alcoholic fatty liver disease (MAFLD) is the most common chronic liver disease, with an estimated prevalence of between 20 and 30% worldwide [1]. MAFLD presents across a spectrum of disease severity, ranging from simple steatosis (SS), through to steatohepatitis steatohepatitis and is a major risk factor for cirrhosis and hepatocellular carcinoma [2,3,4]. Independent of other risk factors, MAFLD increases all-cause mortality by an average of 11.7% (hazard ratio (HR) 1.93 (1.86–2.00)) [5]. The impact of MAFLD on mortality increases with increasing disease severity and ranges from 8.3% (HR 1.71 (1.64–1.790) for SS up to 18.4% (HR 2.44 (2.22–2.69)) for steatohepatitis with fibrosis. The capacity to treat MAFLD diminishes with increasing disease severity. Targeted weight loss of 7 to 10% slows disease progression in mild disease but is less effective in moderate to severe disease [6].

In addition to the direct challenge of treating MAFLD, the disease impacts the treatment of comorbid conditions by perturbing the abundance and activity of hepatic drug metabolising enzymes (DMEs) and transporters resulting in variability in drug exposure. In this regard cytochromes P450 (CYP) are a superfamily of isoenzymes that catalyse the oxidative metabolism of endogenous and exogenous small molecules including drugs. Primarily located in the liver, CYP play a major role in the clearance of >70% of drugs [7] and are considered to be the most important family of DMEs. While the CYP superfamily comprises more than 60 different isoenzymes, CYP 1A2, 2C9, 2C19, 2D6 and 3A4, are the major contributors to drug metabolism [8]. Supporting the observational reports of altered drug exposure in patients with MAFLD, multiple studies conducted in in vitro and pre-clinical animal models of MAFLD have demonstrated differences in CYP protein abundance and activity [9,10,11,12]. Notably the impact of MAFLD on protein abundance and activity in these models differs in terms of both the magnitude and direction of effect between CYP isoenzymes. Despite the prevalence of MAFLD and substantial in vitro and pre-clinical animal model data demonstrating that this disease variably impacts drug exposure, there are limited data evaluating the impact of MAFLD on drug exposure in humans.

Physiologically based pharmacokinetic (PBPK) modelling and simulation is recognised by all major regulators including the US Food and Drug Administration (FDA) [13,14] and European Medicines Agency (EMA) [15,16] as a platform to support regulatory assessment, dosing decisions, and the use of medicines in special populations. PBPK combines physiological data from a population of interest (population profile) with physiochemical and in vitro data for a drug of interest (compound profile) to simulate the exposure of a drug under a specified set of conditions (trial design). This approach has been utilised to describe differences in drug exposure associated with race, sex and various diseases including cancer and rheumatoid arthritis [17,18,19,20]. The primary objective of the current study was to undertake a comprehensive literature review and meta-analysis to define physiological and metabolic changes associated with MAFLD. These data were used to construct a PBPK profile describing a MAFLD disease population. The MAFLD population profile was applied in simulations to predict the impact of disease on exposure to probe substrates for each of the major CYP involved in drug metabolism.

## 2. Results

### 2.1. Construction of MAFLD Population Profile

A summary of the meta-analysis pooling observed changes in CYP protein abundance that informed the MAFLD population profile are presented in Supplemental Table S1. Reported data were combined to determine a geometric mean proportional difference in CYP protein abundance between MAFLD and healthy controls that was applied to scale corresponding parameters in the healthy population (Sim-Healthy Volunteer) profile.

Marked heterogeneity was observed in both the impact of MAFLD on the abundance of different CYP enzymes within a single study, and the impact of MAFLD on the abundance of a specific CYP enzyme across different studies. By way of example, the geometric mean (range) proportional difference in CYP protein abundance between MAFLD and healthy controls ranged from 0.72 (1.0 to 0.44) for CYP2C9 to 0.36 (0.46 to 0.24) for CYP1A2. Accordingly, the CYP2C9 protein abundance in the Sim-Healthy Volunteer population was reduced by 28% in the MAFLD population profile, while the CYP1A2 protein abundance was reduced by 63% (Supplemental Table S1).

Haematocrit and BMI are also physiological parameters associated with differences in drug exposure that are reported to be perturbed in MAFLD patients. Haematocrit was set as 48.2% in males and 42.6% in females in the MAFLD population profile based on an observed mean 1.12-fold increase in this parameter in MAFLD patients compared to healthy controls [21,22] (Supplemental Table S2). Based on a meta-analysis of reported data, mean BMI values were set at 29.7 and 27.3 for male and female subjects in the MAFLD population profile, respectively [21,22,23,24]. Using these BMI inputs, the relationship between height and weight in male and female MAFLD subjects was compared to matched healthy control (Sim-Healthy Volunteer) populations.

### 2.2. Simulated Drug Exposure in MAFLD Population

**Figure 1:**
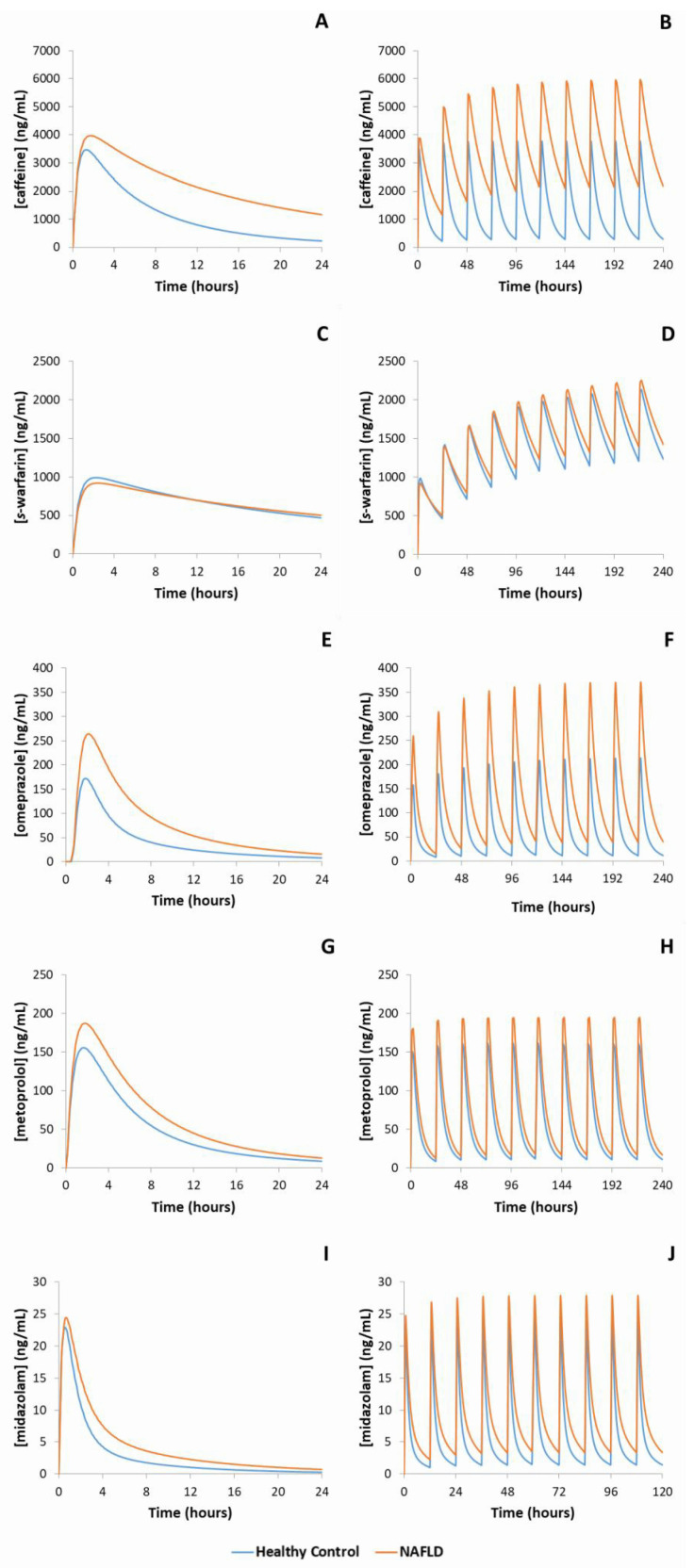
Representative mean simulated concentration-time profiles of probe substrates metabolised by each of the major cytochrome P450 enzymes. Blue line represents healthy control population, orange line represents MAFLD populations. (**A**): caffeine (CYP1A2), single dose; (**B**): caffeine, multiple doses; (**C**): s-warfarin (CYP2C9), single dose; (**D**): s-warfarin, multiple doses; (**E**): omeprazole (CYP2C19), single dose; (**F**): omeprazole, multiple doses; (**G**): metoprolol (CYP2D6), single dose; (**H**): metoprolol, multiple doses, (**I**): midazolam (CYP3A4), single dose; (**J**): midazolam, multiple doses.

The geometric mean (95% CI) simulated AUC and Cmax defining exposure for CYP 1A2, 2C9, 2C19, 2D6 and 3A4 probe substrates in MAFLD and healthy populations are reported in Table 1 and visualized in Figure 1. The difference in probe substrate exposure between MAFLD and healthy controls is presented as a parameter ratio (AUC and Cmax) of MAFLD/healthy control.

For CYP1A2, simulations demonstrated that caffeine AUC was 2.1-fold higher in MAFLD subjects compared to healthy controls following a single oral dose and was 2.9-fold higher in MAFLD subjects when caffeine was dosed to steady state. Likewise, simulated clozapine AUC was 1.8- and 2.2-fold higher in MAFLD subjects compared to healthy controls following single and multiple doses, respectively. Simulated caffeine and clozapine Cmax were also increased in MAFLD subjects compared to healthy controls following single and multiple doses, albeit to a lesser extent. Despite CYP2C9 protein abundance being 28% lower in MAFLD subjects compared to healthy controls, simulations demonstrated essentially no difference (<10%) in either *s*-warfarin or rosiglitazone AUC and Cmax between MAFLD and healthy subjects following either single or multiple doses. Simulations considering the impact of MAFLD on CYP2C19 demonstrated 2.5- and 3.2-fold higher omeprazole AUCs in MAFLD subjects compared to matched healthy controls following single and multiple doses, respectively. When considering CYP2D6, simulated dextromethorphan AUC was 1.3-fold higher in MAFLD subjects compared to controls following a single dose, and 1.4-fold higher in MAFLD subjects following multiple doses. Similarly, following single and multiple doses of metoprolol the simulated AUC was 1.4-fold higher in NALFD subjects compared to healthy controls. Finally, for CYP3A4, simulations demonstrated a 1.6-fold higher single dose midazolam AUC in MAFLD subjects compared to matched healthy controls, and a 1.6-fold higher steady state AUC in MAFLD subjects following multiple doses of midazolam.

### 2.3. Validation of the MAFLD Population Profile

#### 2.3.1. Activity in Human In Vitro Models as Comparator

A summary of the observed changes in in vitro CYP activity and a description of the corresponding study parameters are presented in Table 2. Reported pooled data used to determine a geometric mean proportional difference in CYP activity between in vitro MAFLD and control model systems support reduced activity for CYP 1A2, 2C9, 2C19 2D6 and 3A4. However, like changes in CYP protein abundance, marked heterogeneity was observed for the impact of MAFLD on CYP protein activity within and across studies. By way of example, the mean (range) difference in CYP protein abundance between MAFLD and healthy controls ranged from 0.96 (1.53 to 0.40) for CYP2C9 to 0.42 (0.62 to 0.21) for CYP2C19.

Reported in vitro CYP activities and simulated probe substrate AUCs in healthy and MAFLD subjects are reported in Table 3. Strong concordance between the simulated difference in exposure between MAFLD and healthy controls and the observed difference in in vitro CYP activity between MAFLD and control models was observed for all enzymes. The MFE between simulated and observed MAFLD to healthy control parameter ratios for CYP 1A2, 2C9, 2C19, 2D6 and 3A4 were invariably within the range 1.00 to 1.31.

#### 2.3.2. Activity in Pre-Clinical Animal Models as Comparator

Pharmacokinetic parameters (AUC and Cmax) determined following dosing of animals with selective probe substrates for CYP 1A2, 2C9, 2C19, 2D6 and 3A4 are summarised in Table 4. Increases in AUC and Cmax were consistently reported in MAFLD animals compared to matched healthy controls. Model performance was defined based on the concordance between the observed AUC ratio in MAFLD versus healthy animals compared to simulated AUC ratio in MAFLD versus healthy trial populations.

Strong concordance between the simulated and observed impact of MAFLD was seen for CYP1A2; the MAFLD to healthy AUC ratio for caffeine was 1.3-fold higher in the animal model compared to the simulated population, while the MAFLD to healthy AUC ratio for clozapine was 1.5-fold higher in simulations. Strong concordance was also observed with the CYP3A4 substrate midazolam; the MFE between the simulated and observed MAFLD to healthy AUC ratios was 1.00. There was robust concordance between simulated and observed impact of MAFLD on CYP2D6 and CYP2C9; the MFE between the simulated and observed MAFLD to healthy AUC ratio for metoprolol (CYP2D6), dextromethorphan (CYP2D6) and rosiglitazone (CYP2C9) were 1.57, 2.04 and 2.08, respectively. Except for clozapine, simulations predicted a lesser impact of MAFLD on probe substrate AUC compared to the observed animal model. In contrast, poor concordance between the simulated and observed impact of MAFLD was seen for CYP2C19; the simulated MAFLD to healthy control AUC ratio (2.52) was 4.4-fold lower than the MAFLD to healthy control AUC ratio (11.1) observed in the animal model.

## 3. Discussion

Here, we report the development and validation of a PBPK model describing the physiological and molecular changes observed in patients with MAFLD. This model may be applied to predict changes in drug distribution and clearance for medicines primarily cleared by CYP catalysed metabolism. In recent years, multiple reviews have highlighted the potential value of utilising PBPK modelling and simulation to predict the impact of MAFLD on drug exposure [31,32]. Indeed a prior in vitro mechanistic study interrogating the transcriptional regulation of altered CYP3A4 expression and activity in MAFLD patients utilised PBPK modelling to extrapolate the clinical importance of their findings specifically for drugs primarily cleared by CYP3A4 catalysed metabolism [28].

The PBPK model described here used the same modelling approach as Jamwal [28] for a broader panel of CYP to develop a model that is capable of predicting changes in exposure to a more diverse array of drugs, including those where multiple CYP contribute to hepatic clearance. The model also incorporates changes in body composition and physiological parameters (renal function, haematocrit, plasma protein abundance) to enable consideration of additional factors influencing drug exposure beyond hepatic clearance. The association between MAFLD and alterations in CYP abundance and activity have been extensively studied using in vitro cell/tissue models and in vivo pre-clinical animal models. In contrast, human data are scarce and capacity to support clinical translation is lacking [33]. Here, we demonstrate that perturbations in CYP abundance caused by MAFLD can be reliably extrapolated to the clinical setting through PBPK modelling to predict changes to drug exposure among MAFLD patients. Changes in the concentration time profiles for probe substrates of CYP 1A2 (caffeine and clozapine), 2C9 (s-warfarin and rosiglitazone), 2C19 (omeprazole), 2D6 (dextromethorphan and metoprolol) and CYP3A4 (midazolam) were reliably simulated using the MAFLD population profile.

The performance of the MAFLD PBPK profile was established based on the concordance with available models of MAFLD. Consistent strong concordance was observed between the simulated difference in probe substrate AUC between MAFLD and healthy control populations and the observed difference in activity between in vitro human MAFLD and healthy control models (MFE ≤ 1.31 for all CYP; Table 3). Importantly, the in vitro CYP abundance data that underpin the MAFLD population profile and the in vitro activity data that were used to verify the model performance were derived from independent reports. In contrast to the strong concordance between the simulated probe substrate AUC and activity observed in human in vitro models of MAFLD, variable concordance was seen when comparing simulation data to in vivo pre-clinical animal models of MAFLD. Robust concordance was observed for CYP 1A2, 2D6 and 3A4, with average simulated/observed MFEs for MAFLD to control probe substrate AUC ratios of 1.44, 1.80 and 1.00, respectively. Borderline concordance between the simulated and observed MAFLD to healthy control AUC ratio was seen for CYP2C9 (MFE of 2.08), and poor concordance between the simulated and observed MAFLD to healthy control AUC ratio was seen for CYP2C19 (MFE of 4.40). Notably the poor concordance between the simulated MAFLD to healthy control AUC ratio for omeprazole (CYP2C19 probe) and the corresponding AUC ratio observed in the animal model is driven by an atypically high AUC ratio (11.1) in the animal model that is also not consistent with observed in vitro human data for this enzyme (see Table 3 and Table 4). Taken together, these data demonstrate that overall the MAFLD population profile faithfully reflects the impact of this disease on exposure to drugs that are predominantly cleared by different CYP enzymes.

Applying bioequivalence criteria, a meaningful difference in drug exposure among MAFLD patients was defined as a >20% increase or decrease in the geometric mean AUC compared to healthy controls [34]. Simulations demonstrated that both the single dose and steady state AUCs for caffeine, clozapine, omeprazole, metoprolol, dextromethorphan and midazolam were increased by >20% in the MAFLD population compared to the healthy control population (Table 1). These findings indicate that MAFLD patients are likely to experience meaningfully higher exposure to drugs that are primarily metabolized by CYP 1A2, 2C19, 2D6 and 3A4. Closer monitoring of MAFLD patients using drugs primarily cleared by these CYP, particularly CYP 1A2, 2C19 and 3A4, may be required as the greater drug exposure will increase the risk of toxicity in this population. In contrast, the data from PBPK simulations, in vitro activity assays and pre-clinical animal models indicate that observed differences in CYP2C9 protein abundance among MAFLD patients are unlikely to result in meaningful differences in exposure for drugs primarily cleared by this enzyme. However, inconsistencies in pre-clinical data preclude definitive interpretation of the likelihood of MAFLD induced changes in CYP2C9 activity, and significant changes in exposure to drugs metabolised by this enzyme cannot be excluded. While modelling presented here provides some insight, human trials would provide more conclusive evidence in the absence of consistent preclinical data.

MAFLD is among the causes of chronic liver disease and cirrhosis that have traditionally been broadly categorized by the Child-Pugh scoring system to determine the degree of hepatic impairment. However, the unique alterations to abundance and activity of DMEs and other physiological variables described in NALFD patients do not necessarily correlate with this clinical score when considering drug metabolism and the requirement for dose adjustment [35]. Fortunately, PBPK modelling represents a powerful tool for predicting drug pharmacokinetics in specific populations, offering a promising platform for extrapolating to the clinical setting what we already know about the metabolic changes in MAFLD at the pre-clinical level. It has the potential to inform drug dosing, increase efficiency in drug discovery and development, and even reduce or replace animal studies and clinical trials [36].

The two main limitations of the current study were the inability of the model to discriminate between different MAFLD stages and the inability to account for changes in non-CYP mediated clearance pathways. Sufficient in vivo data regarding perturbation of CYP activity and drug exposure in human subjects with MAFLD is lacking. Some insight is provided in a study of SS and steatohepatitis patients by Woolsey et al., which detected 2.4-fold higher plasma midazolam concentration, compared to controls [30]. This study distinguished between stages of MAFLD and highlight the decline in CYP abundance and activity with disease progression. Indeed, MAFLD has traditionally been considered a spectrum of histological progression from SS to steatohepatitis, however it is also argued that these may actually be two distinct and non-linear disease entities [2]. Considering the pathological heterogeneity that exists amongst the MAFLD population and the absence of effective non-invasive diagnostic techniques, the integration of data from across disease stages was chosen to construct the simulated MAFLD population profile to represent these challenges and provide simpler clinical translation. Furthermore, it is well known that many non-CYP proteins (e.g., conjugating enzymes and drug transporters) contribute to the clearance on many drugs [37]. Further, for some drugs primarily metabolised by CYP such as s-warfarin and tolbutamide [38,39], enzyme-transporter interplay is likely important in determining exposure. Unfortunately, in vivo and in vitro human and animal data regarding the impact of MAFLD on the expression and activity of non-CYP mediated clearance pathways are both lacking and inconsistent. As such these data did not form a sufficient evidence base to inform the MAFLD population profile. Altered protein abundance for some drug transporters in steatohepatitis patients has been reported to result in increased exposure to morphine glucuronide [40]. However, these data are contradicted by data demonstrating that apixaban and rosuvastatin exposure is not altered in MAFLD patients [33]. Further elucidation of these more complex metabolic changes in MAFLD, beyond CYP-mediated metabolism are warranted.

In conclusion, the novel MAFLD population profile reported here robustly integrates preclinical and clinical data to demonstrate meaningful increases in drug exposure that is likely to be relevant across a myriad of therapeutic drug classes. This model has utility in guiding dosing among MAFLD patients to minimize the risk of toxicity in this patient population. Further human studies directly evaluating drug disposition in MAFLD patients are required to corroborate simulated findings, in conjunction with further observations in human tissue and animal models beyond CYP mediated clearance pathways.

## 4. Methods

### 4.1. Construction of MAFLD Population Profile

#### 4.1.1. Population Profile

Physiologically based pharmacokinetic modelling and simulation was performed using SimCYP version 19.1 (Certara, New Jersey, Princeton USA). The MAFLD population profile was constructed by adapting demographic and physiological covariates from the Sim-Healthy population profile based on a meta-analysis of observed data from the published literature.

#### 4.1.2. Substrate Profiles

Simulations were performed using validated in-built substrate profiles for caffeine (Sim-caffeine), clozapine (Sim-clozapine), dextromethorphan (SV-dextromethorphan), metoprolol (SV-metoprolol), midazolam (Sim-midazolam), omeprazole (SV-omeprazole), rosiglitazone (Sim-rosiglitazone), s-warfarin (Sim-s-warfarin) [41].

#### 4.1.3. CYP Protein Abundance

A comprehensive literature review and meta-analysis was conducted to identify all studies describing in vitro and pre-clinical model changes in CYP1A2, 2C9, 2C19, 2D6 and 3A4 protein abundance associated with MAFLD. The analysis included studies reporting changes in CYP abundance based on observations with cultured hepatocytes, liver microsomes and liver biopsy. Data were derived from both human and animal models. Sensitivity analyses were performed to evaluate the impact of sample type and species on the observed effect of MAFLD. The geometric mean proportion to which protein abundance of each CYP is increased or decreased in MAFLD relative to healthy was calculated and used to determine absolute abundance for the relevant MAFLD phenotypes of each CYP (expressed as picomoles/mg protein) (Supplemental Table S1).

#### 4.1.4. Other Parameters

Other physiological parameters affecting drug exposure, including haematocrit, albumin concentration and creatinine concentration, that have been reported to change in MAFLD compared to healthy subjects were also included in the literature review and meta-analysis, and were parameterised for the MAFLD population profile (Supplemental Table S2). Body-mass index (BMI) was independently adjusted for males and females in the MAFLD population profile based on observed data [21,22,23,24]. Adjustments were made by modelling weight versus height using the exponential function described by Cheeti et al. [17]:

$$f=\exp\left(a+x*x_{0}\right)$$

where f is weight; x is height, a and $x_{0}$ are exponent parameters, intercept and slope, respectively. Mathematical models in SimCYP describing height-weight relationships for the Sim-Healthy Volunteer population were adapted for the MAFLD population profile according to these exponent parameters, where a = C0 and $x_{0}$ = C1 and where C0 and C1 are scaling co-efficients on SimCYP. Calculations are summarized in Supplemental Table S3.

### 4.2. Simulated Drug Exposure in MAFLD Population

Virtual clinical trials were performed to simulate differences in exposure to the selective probe substrates midazolam (5 mg PO; CYP3A4), caffeine (150 mg PO; CYP1A2), s-warfarin (10 mg PO; CYP2C9), dextromethorphan (30 mg PO; CYP2D6), and omeprazole (20 mg PO; CYP2C19) between healthy subjects and subjects with MAFLD.

Simulations were performed using built-in compound (substrate) profiles, with trial cohorts derived from the novel MAFLD population and Sim-Healthy Volunteer populations. The proportion of females in the trial cohorts was 0.5 and age range was 20 to 65 years. Simulations comprising 20 trials with 10 subjects per trial (*n* = 200) were conducted for each probe substrate to determine the concentration time profile following orally administered single and multiple (*n* = 10) doses. For single dose trials the area under the plasma concentration time curve (AUC) and maximal plasma concentration (Cmax) for each probe substrate was simulated over 24 h following a single oral dose. For multiple dose trials the steady state AUC and Cmax for each probe substrate was simulated over 24 h following the last oral dose. In multiple dose trials probe substrates were dosed once every 24 h, except for midazolam and dextromethorphan, which were dosed every 12 h. The simulated geometric mean (95% confidence intervals) AUC and Cmax following single and multiple doses of each probe substrate were compared between the MAFLD and healthy populations and are reported as a MAFLD to healthy ratio.

### 4.3. Validation of the MAFLD Population Profile

The MAFLD population profile was verified using two orthogonal approaches; as the profile was built using exclusively protein abundance data, the primary approach used to verify the profile was comparison of simulated and observed metabolic activities for each CYP enzymes.

A comprehensive literature review was conducted to identify studies comparing in vitro metabolic activities for CYP 1A2, 2C9, 2C19, 2D6 and 3A4 between healthy and MAFLD models. Specifically, activity data was obtained from in vitro studies, performed using probe substrate assays with cultured human hepatocytes, human liver microsomes and human hepatocytes obtained from healthy and MAFLD tissue samples. The geometric mean proportion to which the in vitro activity for each CYP was increased or decreased in the MAFLD model relative to healthy controls was calculated and used as observed comparator against which the accuracy of the simulated activity (MAFLD to Sim-Healthy Volunteer AUC ratio) was evaluated for each probe substrate. Activity data was also obtained from studies in pre-clinical animal models that reported AUC and Cmax values for probe substrates in MAFLD animals compared to healthy control animals. The geometric mean ratio of AUC and Cmax in MAFLD relative to healthy control for each probe substrate was used as observed comparator against which the accuracy of simulated data was evaluated.

Additional simulations were performed using built-in compound profiles for clozapine (12.5 mg PO), rosiglitazone (4 mg PO) and metoprolol (100 mg PO) to allow for direct comparison between simulated and reported exposure profiles. These simulations were used as a secondary approach to verify the MAFLD population model. There was insufficient data from human clinical trials for each of the selective probe substrates to include as a comparator when validating the profile. Model performance was evaluated based on the concordance of the simulated MAFLD to healthy control AUC ratio with the observed MAFLD to healthy control parameter ratio in the in vitro and pre-clinical animal models. An absolute mean fold error (MFE) between the simulated and observed parameter ratios within 2-fold was considered robust performance.

## Figures and Tables

**Table 1:** Simulated geometric mean (95% CI) pharmacokinetic parameters defining probe substrate exposure in MAFLD and healthy control populations following single and multiple oral doses.

Probe Substrate (Enzyme)	Population	Single Dose		Multiple Doses	
		Cmax (ng/mL)	AUC (ng/mL.hr)	Cmax (ng/mL)	AUC (ng/mL.hr)
Caffeine (CYP1A2)	Healthy	3399 (2190–5481)	23,712 (8761–57,383)	3597 (2208–6105)	25,130 (8774–73,879)
	MAFLD	3885 (2614–6056)	50,331 (19,990–97,084)	5568 (3083–12,856)	72,715 (20,523–244,322)
	Ratio ^	1.14	2.12 ^#^	1.55 ^#^	2.89 ^#^
Clozapine (CYP1A2)	Healthy	54.4 (27.2–108)	436 (209–890)	58.4 (29.4–109)	469 (214–1029)
	MAFLD	62.6 (34.0–127)	788 (407–1567)	81.3 (46.6–164)	1026 (451–2208)
	Ratio ^	1.15	1.81 ^#^	1.39 ^#^	2.19 ^#^
S-Warfarin (CYP2C9)	Healthy	924 (442–1714)	15,458 (7879–28,198)	1877 (801–4499)	31,630 (11,056–101,358)
	MAFLD	866 (484–1593)	15,500 (8577–30,203)	1983 (897–5000)	35,778 (12,803–106,346)
	Ratio ^	0.94	1.00	1.06	1.13
Rosiglitazone (CYP2C9)	Healthy	245 (165–346)	1152 (385–2378)	248 (166–352)	1166 (385–2448)
	MAFLD	241 (157–366)	1331 (484–3169)	247 (158–382)	1364 (484–3366)
	Ratio ^	0.98	1.16	0.99	1.17
Omeprazole (CYP2C19)	Healthy	153 (58.0–444)	467 (110–4505)	191 (62.0–503)	666 (120–5130)
	MAFLD	236 (101–509)	1174 (253–5528)	323 (125–777)	2112 (355–10,090)
	Ratio ^	1.54 ^#^	2.52 ^#^	1.70 ^#^	3.17 ^#^
Dextromethorphan (CYP2D6)	Healthy	4.25 (1.18–20.9)	49.9 (14.3–393)	7.56 (2.03–85.2)	63.6 (15.8–965)
	MAFLD	5.08 (1.57–18.7)	66.0 (20.8–383)	10.1 (2.96–104)	89.8 (26.4–1170)
	Ratio ^	1.20 ^#^	1.32 ^#^	1.33 ^#^	1.41 ^#^
Metoprolol (CYP2D6)	Healthy	1412 (63.9–366)	841 (257–4131)	145 (64.1–385)	863 (257–4685)
	MAFLD	172 (85.7–353)	1223 (425–4208)	180 (86.2–410)	1276 (425–5093)
	Ratio ^	1.22 ^#^	1.45 ^#^	1.24 ^#^	1.48 ^#^
Midazolam (CYP3A4)	Healthy	18.7 (5.75–53.6)	54.7 (14.9–151)	19.9 (5.89–57.2)	56.1 (15.2–153)
	MAFLD	20.7 (8.84–63.5)	84.8 (23.7–268)	23.4 (9.09–64.3)	89.7 (23.8–274)
	Ratio ^	1.11	1.55 ^#^	1.18	1.60 ^#^

^ Ratio: geometric mean MAFLD parameter/geometric mean healthy control parameter; ^#^ Indicates lack of equivalence between MAFLD and healthy control populations (parameter ratio > 1.2).

**Table 2:** Observed geometric mean differences in the in vitro metabolic activity of cytochrome P450 enzymes in MAFLD versus healthy control models.

Enzyme	Probe Substrate Reaction	Difference ^	Ref	Geometric Mean Difference ^
CYP1A2	7-methoxyresorufin O-demethylation	0.46	[25]	0.52
	Phenacetin O-deethylation	0.58	[26]	
CYP2C9	Diclofenac 4′-hydroxylation	0.82	[25]	0.96
	Testosterone 16ß-hydroxlation	0.64	[25]	
	Testosterone 16ß-hydroxlation	0.40	[27]	
	Diclofenac 4′-hydroxylation	1.53	[26]	
	Tolbutamide 4-hydroxylation	1.42	[26]	
CYP2C19	Androstenedione	0.46	[25]	0.42
	Testosterone 16ß-hydroxlation	0.40	[27]	
	Androstenedione	0.62	[27]	
	Mephenytoin 4′hydroxylation	0.21	[26]	
CYP2D6	Dextromethorphan O-demethylation	0.68	[26]	0.68
CYP3A4	Midazolam 1′-hydroxylation	0.45	[28]	0.49
	Testosterone 6ß-hydroxlation	0.55	[25]	
	Testosterone 6ß-hydroxlation	0.57	[25]	
	Testosterone 2ß-hydroxlation	0.41	[25]	
	Testosterone 15ß-hydroxlation	0.55	[27]	
	Testosterone 6ß-hydroxlation	0.44	[27]	
	Testosterone 2ß-hydroxlation	0.43	[27]	
	Testosterone 6ß-hydroxlation	0.46	[29]	
	Midazolam 1′-hydroxylation	0.41	[28]	
	Midazolam 1′-hydroxylation	0.61	[30]	

^ Difference = Activity in MAFLD model/activity in healthy control model.

**Table 3:** Simulated and observed parameter ratios defining the difference in cytochrome P450 enzyme activity between MAFLD and healthy control models.

Enzyme	Observed In Vitro Activity Ratio ^	Simulated Probe Substrate AUC Ratio ^	Absolute Mean Fold Error ^#^
CYP1A2	1.93	2.12	1.10
CYP2C9	1.04	1.00	1.04
CYP2C19	2.38	2.52	1.06
CYP2D6	1.46	1.32	1.11
CYP3A4	2.03	1.55	1.31

^ Ratio: geometric mean MAFLD parameter/geometric mean healthy control parameter; ^#^ Absolute Mean Fold Error: Absolute ratio between the simulated activity ratio and observed activity ratio.

**Table 4:** Simulated and observed parameter ratios defining the difference in exposure to probe substrates between MAFLD and healthy controls.

Enzyme	Probe Substrate	Parameter	Observed in Animals	Simulated in Humans	Absolute Mean Fold Error ^#^	Ref
CYP1A2	Caffeine	AUC ratio	2.90	2.12	1.37	[9]
		Cmax ratio	2.50	1.14	2.19	
	Clozapine	AUC ratio	1.20	1.81	1.50	[12]
		Cmax ratio	0.69	1.15	1.66	
CYP2C9	Rosiglitazone	AUC ratio	2.41	1.16	2.08	[10]
		Cmax ratio	0.86	0.982	1.14	
CYP2C19	Omeprazole	AUC ratio	11.1	2.52	4.40	[9]
		Cmax ratio	6.50	1.54	4.23	
CYP2D6	Dextromethorphan	AUC ratio	2.69	1.32	2.03	[9]
		Cmax ratio	3.31	1.20	2.77	
	Metoprolol	AUC ratio	2.28	1.45	1.57	[11]
		Cmax ratio	1.41	1.22	1.16	
CYP3A4	Midazolam	AUC ratio	1.55	1.55	1.00	[9]
		Cmax ratio	1.22	1.11	1.10	

^#^ Absolute Mean Fold Error: Absolute ratio between the simulated activity ratio and observed activity ratio.

## Data Availability

The data that support the findings of this study are available from the corresponding author upon reasonable request.

## Supplementary Materials

The following supporting information can be downloaded at: https://www.mdpi.com/article/10.3390/ijms231911751/s1.
