# Supplementary material for: A Physiologically Based Pharmacokinetic Model to Predict the Impact of Metabolic Changes Associated with Metabolic Associated Fatty Liver Disease on Drug Exposure
Source: Int J Mol Sci. 2022 Oct 4;23(19):11751. doi: 10.3390/ijms231911751 (PMC9570165; doi:10.3390/ijms231911751)
Supplement: Supplementary file 1 [file ijms-23-11751-s001.zip › ijms-1887904-supplementary.pdf]

Supplemental Data: A physiologically based pharmacokinetic model to predict the impact of metabolic changes associated with non-alcoholic fatty liver disease on drug exposure

Elise M Newman, Andrew Rowland

Table S1: Changes in abundance of major cytochrome P450 enzymes in *in vitro* and animal model studies of NAFLD compared to healthy, with proportional change used to calculate CYP abundance (pmol/mg protein) in the PBPK model of NAFLD.

Enzyme	Geometric mean Change in CYP Protein Abundance	Proportional Change^	Ref	Geometric mean Proportional Change^
**CYP1A2**	↓	0.4583	(28)	**0. 3647**
		0.2357	(8)	
		0.4	(37)	
CYP Phenotype	EM	PM	IM	UM
CYP Abundance: Sim-Healthy	52	-	-	-
CYP Abundance: Sim-NAFLD (Calculated)	18.96	-	-	-
**CYP2C9**	↓	1.0	(28)	**0.7231**
		0.4462	(8)	
CYP Phenotype	EM	PM	IM	UM
CYP Abundance: Sim-Healthy	73	29	-	-
CYP Abundance: Sim-NAFLD (Calculated)	52.78	20.97	-	-
**CYP2C19**	↓	0.4172	(28)	**0.4172**
CYP Phenotype	EM	PM	IM	UM
CYP Abundance: Sim-Healthy	1.836	-	-	3.63
CYP Abundance: Sim-NAFLD (Calculated)	5.5	-	-	10.88
**CYP2D6**	↓	0.7037	(28)	**0.7214**
		0.7391	(8)	
CYP Phenotype	EM	PM	IM	UM
CYP Abundance: Sim-Healthy	9.4	-	-	18.8
CYP Abundance: Sim-NAFLD (Calculated)	6.781	-	-	13.56

**CYP3A4**	↓	0.8593	(28)	**0.6393**
		0.4852	(27)	
		0.6778	(27)	
		0.7059	(25)	
		0.7191	(8)	
		0.478	(38)	
CYP Phenotype	EM	PM	IM	UM
CYP Abundance: Sim-Healthy	137	-	-	-
CYP Abundance: Sim-NAFLD (Calculated)	87.59	-	-	-

Table S2: Changes in other physiological characteristics in human subjects with NAFLD compared to healthy, with proportional change used to calculate values in the PBPK model.

Parameter	Proportional Change (NAFLD/Healthy)	Ref	Geometric mean Proportional Change
**Haematocrit**	1.095	(16)	**1.12**
	1.145	(17)	
		Male	*Female*
	Sim-Healthy (SimCYP) (%)	43	38
	Sim-NAFLD (Calculated) (%)	48.2	42.6
**Albumin**	Unchanged	(19)	
		(18)	
**Creatinine**	Unchanged	(19)	
		(39)	

Table S3: Modelling of relationship between height and weight in healthy and NAFLD populations.

	Healthy: Male		Healthy: Female		NAFLD: Male		NAFLD: Female	
					BMI = 29.7[$]		BMI = 27.3[#]	
**Height in SimCYP (cm)**	**Weight***	**Weight (ln)**	**Weight***	**Weight (ln)**	**Weight^**	**Weight (ln)**	**Weight^**	**Weight (ln)**
148	60.837	4.108	59.448	4.085	64.973	4.174	59.798	4.091
153	63.924	4.158	62.215	4.131	69.437	4.240	63.907	4.157
158	67.168	4.207	65.111	4.176	74.075	4.305	68.152	4.222
163	70.577	4.257	68.142	4.222	78.810	4.367	72.533	4.284
168	74.158	4.306	71.315	4.267	83.719	4.427	77.052	4.344
173	77.921	4.356	74.634	4.313	88.777	4.486	81.706	4.403
178	81.876	4.405	78.109	4.358	93.983	4.543	86.497	4.460
**Equation from graph of weight(ln) against height**	***Y = 0.0099x + 2.634***		***Y = 0.0091x + 2.7383***		***Y = 0.0123x + 2.3589***		***Y = 0.0123x + 2.2759***	
**SimCYP Co-efficient: C0 (= a = intercept)**	2.634		2.7383		2.3589		2.2759	
**SimCYP Co-efficient: C1 (= x_0 = slope)**	0.0099		0.0091		0.0123		0.0123	

$ Male BMI calculated as geometric mean of reported (16-19); # Female BMI reported (18); * Weights calculated from equation weight = exp(a + x + x0) where a = intercept, x = height and x0 = slope; ^ Weights calculated from reported BMI and heights obtained from SimCyp using equation BMI = kg/m^2
